# Conceptualizing and Managing Medical Emergencies Where No Formal Paramedical System Exists: Perspectives from a Remote Indigenous Community in Canada

**DOI:** 10.3390/ijerph15020267

**Published:** 2018-02-04

**Authors:** Jeffrey Curran, Stephen D. Ritchie, Jackson Beardy, David VanderBurgh, Karen Born, John Lewko, Aaron M. Orkin

**Affiliations:** 1 Northern Ontario School of Medicine, Thunder Bay, ON P7B 5E1, Canada; david.vanderburgh@nosm.ca; 2 Centre for Rural and Northern Health Research, Laurentian University, Sudbury, ON P3E 2C6, Canada; sritchie@laurentian.ca (S.D.R.); aorkin@nosm.ca (A.M.O.); 3 Sachigo Lake First Nation, Unorganized Kenora District, ON P0V 2P0, Canada; jacksonabeardy@sachigo.ca; 4 Institute for Health Policy, Management & Evaluation, Faculty of Medicine, University of Toronto, Toronto, ON M5T 3M6, Canada; karen.born@utoronto.ca; 5 School of Rural and Northern Health, Laurentian University, Sudbury, ON P3E 2C6, Canada; jlewko@laurentian.ca; 6 Department of Family and Community Medicine, University of Toronto, Toronto, ON M5G 1V7, Canada; 7 Schwartz/Reisman Emergency Medicine Institute, Mt. Sinai Hospital, Toronto, ON M5G 1X5, Canada

**Keywords:** Indigenous health, pre-hospital care, emergency, community-based emergency care

## Abstract

(1) Background: Remote communities in Canada lack an equitable emergency medical response capacity compared to other communities. Community-based emergency care (CBEC) training for laypeople is a model that has the potential to enhance the medical emergency response capacity in isolated and resource-limited contexts. The purpose of this study was to understand the characteristics of medical emergencies and to conceptualize and present a framework for what a medical emergency is for one remote Indigenous community in northwestern Ontario, in order to inform the development of CBEC training. (2) Methods: This study adhered to the principles of community-based participatory research and realist evaluation; it was an integrated component of the formative evaluation of the second Sachigo Lake Wilderness Emergency Response Education Initiative (SLWEREI) training course in 2012. Twelve members of Sachigo Lake First Nation participated in the training course, along with local nursing staff, police officers, community Elders, and course instructors (n = 24 total), who participated in interviews, focus groups, and a collaborative discussion of local health issues in the development of the SLWEREI. (3) Results: The qualitative results are organized into sections that describe the types of local health emergencies and the informal response system of community members in addressing these emergencies. Prominent themes of health adversity that emerged were an inability to manage chronic conditions and fears of exacerbations, the lack of capacity for addressing mental illness, and the high prevalence of injury for community members. (4) Discussion: A three-point framework of what constitutes local perceptions of an emergency emerged from the findings in this study: (1) a sense of isolation; (2) a condition with a potentially adverse outcome; and (3) a need for help.

## 1. Introduction

Remote communities in Canada face a disproportionate burden of health emergencies and a lack of emergency medical services [1,2,3]. Community-based emergency care (CBEC) training is a model that has the potential to reduce inequities in prehospital care in isolated communities [4]. Emergency care training for laypersons increases helping behavior [5] and recognition of mental health issues [6], and decreases response times and mortality rates in many resource limited areas [7,8]. Emergency care training programs must be tailored to specific community contexts to build capacity effectively. Conventional first aid training contains language, pedagogy, and curriculum that is often inappropriate for remote Indigenous communities that do not have a formal response system for medical emergencies [9]. Health leaders in remote northern Ontario communities have confirmed the need for a community-based solution to existing service gaps, and identified four issues related to the current state of emergency response systems: (1) inequity in response capacity and services; (2) lack of formalized dispatch systems; (3) turnover and burnout in volunteer emergency services; and (4) challenges related to first aid training [3].

The purpose of this paper is to present how people in one remote Indigenous community in northwestern Ontario understand and conceptualize medical emergencies, and to offer an analysis of how local notions of emergencies might inform the development of local emergency care management systems. An acute health condition is any illness or injury in which the time to treatment should occur within minutes or hours to reduce suffering, morbidity or mortality [10]. However, it is important to focus on perceptions of emergencies rather than a biomedical model of acute health conditions, as layperson perception of what an emergency medical condition means is diverse [11], and biomedical models ignore the meaning and context of a person’s illness. Isolated and remote environments influence local notions of health emergencies, such that the severity of the situation has more to do with location and available resources than medical complexity. Many Indigenous communities are located in remote wilderness contexts in Canada, with no permanent road access and no formal emergency medical services, including emergency dispatch or ambulance services. Emergency care systems and training must consider this local context in concert with unique cultural and historical factors to offer appropriate local solutions and address health disparities.

The Sachigo Lake Wilderness Emergency Response Education Initiative (SLWEREI) was a collaborative effort to develop and train local laypeople to respond to health emergencies in one remote northern Ontario community [12,13]. The SLWEREI was a pilot program designed to assess the ability of lay responders to fill service gaps that exist in remote communities, where paramedics and other professional emergency services are non-existent or not operational [12,13]. This paper is based on the collaborative analysis of a second training course revised from the original SLWEREI model and delivered within the community in 2012 [14].

### Health of Remote Indigenous Peoples in Ontario

**Figure 1:**
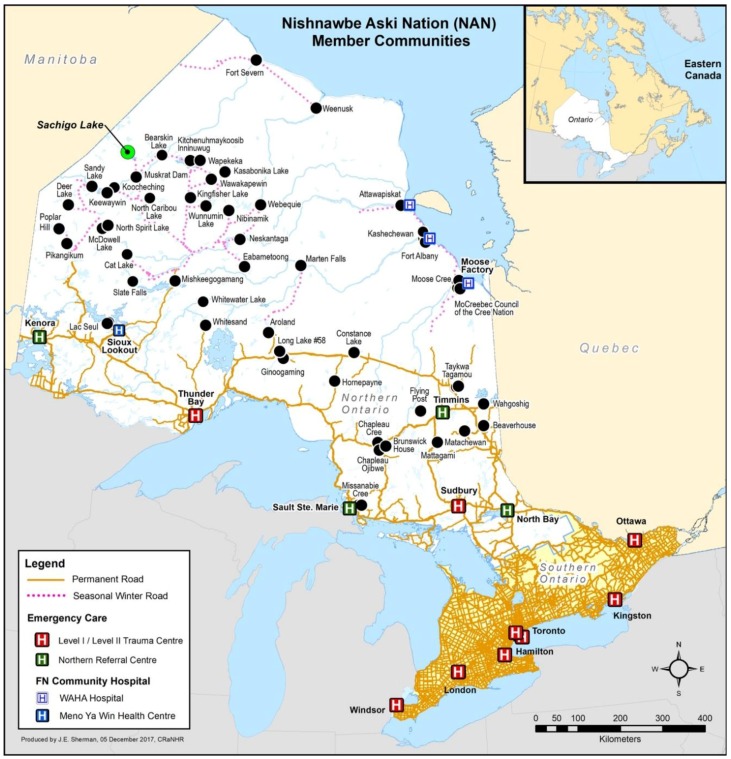
Map of Nishinawbe Aski Nation member communities, road access, community hospitals, and emergency care referral centers in northern Ontario.

Canadians living in remote communities experience higher mortality rates than urban residents [2], with remote environments exacerbating the health impacts related to poorer service availability, higher levels of personal risk, and increasingly hazardous environmental and transportation conditions [15]. There are approximately 55 communities in northern Ontario, Canada that do not have access to an emergency department within 60 minutes travel [16]; 33 of these communities are considered remote—without permanent road access [17]. Many of the remote communities in northern Ontario are Indigenous reserves represented by the Nishnawbe Aski Nation, a political organization of 49 communities encompassing two-thirds of the Ontario landmass [18]. See Figure 1.

Indigenous people in Canada experience a disproportionate burden of poorer health outcomes and double the age-standardized mortality rate compared to the general population [19]. There are elevated rates of chronic conditions such as high blood pressure and heart disease among Indigenous people [20,21] and a fourfold greater risk of severe trauma compared to the general population [22]. Indigenous people on reserves also experience elevated rates of injuries and self-harm [1,23]. The suicide rate among Indigenous youth is seven times higher than non-Indigenous youth and is on the rise [24]. Major barriers to improving the health status of Indigenous people include the unavailability of medical transport, lack of health professionals and facilities, and culturally inappropriate health services [25]. Despite considerable research related to Indigenous health issues, most studies aggregate data on a provincial or national level, which leaves a lack of community-level health data, especially concerning injury and prehospital care [26].

In one area of northwestern Ontario, the Sioux Lookout region, members of remote reserves face injury rates five to eight times that of the general population of Canada, which accounts for 30% of all deaths [27]. Furthermore, it was estimated that as many as 90% of the deaths due to injuries in the Sioux Lookout region occurred before medical care was attained [28]. Despite these alarming trends, there has not been an updated morbidity and mortality profile in this area for over twenty years. The lack of current data and data systems represents an additional injustice and threat to health equity for Indigenous peoples living in remote areas.

Indigenous peoples living in remote areas of Canada should have access to emergency health services and systems that can address existing health inequities, and deliver on universal, comprehensive, and accessible health care under the Canada Health Act Indigenous Treaty rights to health and health care. The Truth and Reconciliation Commission of Canada set forth a call to action to identify and close the gap in health outcomes between Indigenous and non-Indigenous communities, including a specific focus on chronic diseases, injury incidence, and availability of appropriate health services [29]. The development of sustainable and effective emergency medical management systems should be informed by the local context—including local perceptions of what constitutes an emergency—to respond to local needs and introduce programs that build resilience in response to local emergencies and crises. Exploring local perceptions of medical emergencies in Sachigo Lake First Nation was a pivotal part of the development of the SLWEREI. The research questions were: (1) What is the nature of medical emergencies in Sachigo Lake First Nation? and (2) What is the nature of the informal emergency response system in Sachigo Lake First Nation?

## 2. Materials and Methods

This qualitative study followed principles of community-based participatory research, reflecting mutual respect and co-learning between partners, individual and community capacity building, systems change, and balancing research and action [30,31,32]. The study was conducted in the form of a community-based formative program evaluation that was focused on developing a locally specific medical training program for community members. Thus, researchers, course instructors, and local participants worked collaboratively together to deliver, study, and improve the SLWEREI course. The community-based participatory research framework of the study was also informed by complementary principles of realist evaluation [33], which emphasizes the need to understand the program mechanisms and outcomes within the broader social and cultural context that the program is operating in and to explore why, where, and how [34]. Principles of realist evaluation were instrumental in understanding the interplay of program and community [33]. Realist evaluation shaped the sampling strategy, data collection, and analysis through the intentional recruitment of participants that had an intimate knowledge of health issues within the community and program processes; this enabled a more thorough consideration and dialogue of the interplay of program mechanisms within this context. A discussion of the interaction of community-based participatory research and realist evaluation as complimentary methodological frameworks for this study is available elsewhere [33].

### 2.1. Population and Sample

We focused on identifying and recruiting people that have a vested interest in the health and well-being of Sachigo Lake First Nation. The community Health Director (J.B.) had a leadership role in inviting course participants from target groups (teachers, community Band staff, volunteer First Responders, and Canadian Rangers) that were more likely to be involved in first aid situations, as well as members of the community from dispersed locations. Other participants of the course were recruited through posters, radiobroadcasts, and word-of-mouth between community members. Study participants consisted of 12 members of Sachigo Lake First Nation who participated in the 2012 SLWEREI course, two staff from the local nurse-staffed primary care clinic, two staff from the local police detachment, five course instructors, one community Elder external to the course, and two university researchers (total n = 24). Local residents external to the course were included in order to fully understand the health issues among people in the area and impact of the program within the local context. The course instructors provided further insight into pedagogical and medical course processes. This diverse array of participant stakeholders reflected the complementary principles of realist evaluation and participatory research. Age and gender were not criteria in the purposive sampling strategy; however, participants had a relatively wide age range (young adult to senior) and consisted of 14 males and 10 females. The 17 community members of Sachigo Lake who participated in the study represent approximately 3% of the local population [35]. Table 1 summarizes relevant characteristics of Sachigo Lake First Nation.

### 2.2. Data Collection and Collaborative Analysis

Concurrent with the delivery of the SLWEREI course in May 2012, university researchers (and authors) J.C. and S.D.R. conducted 12 interviews (I), six small focus groups with 4–6 course participants, two large focus groups with 12 course participants, and a concluding written survey with nine participants. During this time, J.C. also collected observational notes. Interviews were semi-structured (see Table 2) and adapted freely based on emerging observations. Interviews were conducted with 7 course participants, 5 community members external to the course, and 5 course instructors. Interview and focus group data were collected with digital audio recorders, then transcribed verbatim. All 12 course participants had the opportunity to engage in focus group discussion through a rotation between skill-building activities, simulations, and focus groups during the course. Thus, data analysis was iterative and concurrent with data collection. Emerging themes were discussed with the team conducting the course and research during evening reflective meetings, as well as with the course participants in subsequent interviews and small focus groups. Participants provided input on the emerging themes, including their descriptions and perceived validity, as well as provided input on how to further gather information related to emerging themes. The concluding survey was created based on the preference of and input from the course participants themselves, and nine of the 10 participants that completed the course submitted a written survey. Following the conclusion of the course, J.C. conducted thematic analysis of verbatim quotes using NVivo 9.0 analytic software (QSR International, Melbourne, Australia).

Follow-up data collection occurred in February–April 2013 (J.C.), consisting of phone interviews that were recorded and transcribed, along with informal discussion about the analysis process and emergent themes with four course participants and the five course instructors/researchers. This enabled confirmation and refinement of emergent themes from the analysis, and exploration of perceptions nine months after the course concluded. Members of the research team (A.M.O., D.V., J.C., and M.F.) also returned to Sachigo Lake First Nation to conduct a plain-language community presentation, as well as a knowledge exchange with 13 community members in February 2013. The study was supported by Sachigo Lake Chief and Council, the Nishnawbe Aski Nation, and the Sioux Lookout First Nations Health Authority. Ethical approval was received from the Research Ethics Boards of Lakehead University (REB #008 09-10; 23 January 2012) and Laurentian University (REB #2012-03-03; 30 March 2012).

## 3. Results

The results are organized into two sections that reflect the two research questions: (1) the nature of health emergencies encountered by community members; and (2) the informal response system within the community and in the remote context of traditional land. Dominant themes related to the nature of health emergencies included an inability to manage chronic conditions locally and fears of exacerbations occurring away from help, the emotional burden of mental illness and lack of means to address it, and the high prevalence of injuries that occur in the remote context. The dominant theme related to the informal response system was the contrast and contextual differences between health adversity faced within the community and the experience of people away from the isolated community when help is hours to days away. Community members rely on untrained or undertrained peers to respond to these situations and provide assistance. As the community members and those with personal experience were best suited to describe local health adversity, their voices are highlighted in the results. 

### 3.1. The Nature of Health Emergencies

Chronic conditions. Community members spoke about the inherent difficulty of having chronically ill people within the community with very little in terms of supportive care, transportation, and patient education. Heart disease and diabetes were commonly-reported health conditions affecting community members. Community members reported that diabetes is difficult to avoid and manage in the remote context. Living in a remote community creates challenges in affording and obtaining needed foods and medications to manage health. “Things we’re supposed to eat not available” (9 May, observational notes). Fresh food such as fruits, vegetables, and proteins can be expensive, or inaccessible for many community members. Although diabetes is a chronic condition, it can, and often does, lead to acute health emergencies. “I’ve seen seizures. Almost all of us have” (9 May, observational notes). “…my niece went to check her sister… When she found her, she was on the floor…” (10 May, I 2).

Acute manifestations of chronic health conditions such as heart disease and diabetes present major issues for members of the community to manage in remote locations. For community members, leaving the community to hunt, fish, and gather wood is an important part of their lifestyle and a necessity for living in the area. Community members reflected on a recent situation in which a member of a hunting party had a heart attack while away on a hunting expedition. “All of a sudden, he just flipped over headfirst into the moss. It’s about 50 air miles from here…the spring hunt” (10 May, I 5). “There was no way to dispatch a bush plane in there in the evening because the nearest one is about an hour away or 45 minutes away from here and by the time they would get there it would be dark and there’s no landing light strips up there” (6 May, I 2). “So there was nothing we could do. We tried calling Air Ambulance to see if they wanted a helicopter…this was impossible, so we had to wait until next light…” (7 May, I 1). The hunting party had to deal with the situation themselves, manage the heart attack, and transport the patient back to camp, while keeping him warm in sub-zero temperatures overnight. This instance and many others weigh heavy on the minds of community members, many of whom are living with diabetes and heart disease. Even minor complications can become life threatening while away from the community, yet traveling on the land surrounding the community is integral to the traditional lifestyle of the community.

Mental health and addiction. Mental health and addiction emerged as a theme when discussing health emergencies experienced by community members. Community members discussed the prevalence of alcohol and prescription drug abuse and the impact that it has had: “I think a lot of people suffer from different forms of mental health issues, especially with substance abuse as far as that goes. Like a lot of people try and numb, numb the pain there with different substances” (10 May, I 1). Another community member reflected on the inescapable reality of drug use in the community and the need to address it. “… it’s an epidemic that’s happening throughout and its prescription drug abuse. I see a lot of that… You can’t not see it especially in a tight community like this. And I think it’s something that we should really talk about” (10 May, I 3).

Community members also reflected on the high prevalence of suicide among remote Indigenous communities in the region. “But that’s what’s all over northern Ontario… Because it’s a suicide epidemic happening” (10 May, small focus group 2). “We had a suicide here…. we know everybody around here. Yeah, it was (hard for the community)… I guess they were drinking and something happened…” (10 May, I 3). “…we had like about seven of them all in one month. Attempt(ed) suicides. Yeah, a couple of attempt(ed) hangings, overdoses” (7 May, I 1). Community members discussed the hardships of having friends and family commit suicide resulting in feelings of helplessness. “What do you do if you find someone hanging? It’s happened to lots of people I know” (9 May, observational notes). One community member later reflected that he had lost 10 friends to suicide. These comments illustrate the compelling issue of mental health emergencies in this one region of northern Ontario. Participants felt frustrated and helpless with the prevalence of depression and suicide and their limited means to address it.

Injury. Residents of the community reflected on the substantial health adversity related to injury from events such as physical violence, house fires, snowmobile and all-terrain vehicle accidents, motor vehicle collisions, sports injuries, and slips and falls. People in the community are distributed over a large area; thus, vehicles are necessary for travelling between homes, hunting outside the community, gathering firewood, travelling to ceremonial grounds, and going further out on the land. This frequent travel over rough terrain, coupled with a young and active population, makes injuries a common event. “One time last year, there was a snowmobile accident. …I had to cut the pants just to put pressure on (the wound)… By then he couldn’t walk… If I wasn’t there, you know. What would happen?” (10 May, I 2). When injuries occur, locals depend on peers and passers-by for life-saving care.

Community members also reflected on issues concerning injuries occurring in remote wilderness locations that involve hunting and travelling over rugged terrain. “…accidents happen out there. …dislocated his shoulder while he was lifting up the moose and carrying it out” (10 May, I 3). Often untrained and ill-equipped community members must manage these situations themselves, using only what they have around them. “There are risks that we can get into. …like chainsaw accident or severely cut your leg, or something happens with your snowmobile, especially in the winter time…” (8 May, small focus group 1). The distance involved in transporting people back to the community can amplify the severity of injuries sustained in remote contexts. “And I’ve seen so often where people are brought in un-splinted with often limbs dangling loose…there’s an immense amount of post-injury damage that’s being done just in the moving, like the getting them (to the community nursing station)” (11 May, I 1). Traveling vast distances over rough terrain is a part of the everyday life in a remote community like Sachigo Lake and community members are accustomed to making extended journeys in search of food, supplies, or traveling to nearby communities.

### 3.2. Informal Response System

Within community. The trained healthcare professionals from the nursing station do not provide care outside the nursing station, and there are no emergency dispatch services or formal paramedical services; thus, community members must provide all first aid and patient mobilization. Community members rely on word-of-mouth and telephone calls to each other to activate an informal response system. This consists of sending people to get help on foot, or placing a call to a community member tasked with driving a vehicle that may or may not be equipped with a stretcher. Many community members then arrive on-scene and contribute their knowledge and assistance. Although this system is informal and variable, it is often very timely. “When something happens, the whole community shows up. Everybody… There wouldn’t be just one leader assisting on the scene… The community comes together as one in anything like that” (10 May, I 2). In this fashion, the community shares in the responsibility of helping one another in a medical emergency. Depending on the severity and nature of the medical issue, the person needing assistance is then provided care to the best ability of those around and/or transported to the nursing station to be transferred to the care of the local nursing staff.

The need to mobilize untrained community members and local resources places an emotional burden on locals. The role of care provider is challenging, since the patient is often a friend or family member in the close-knit community. A community member reflected on assisting with cardiopulmonary resuscitation (CPR) within the nursing station with a physician managing the situation by phone: “…then the doctor said there’s nothing more we can do. You guys did everything. You have to stop. And I kept on going, looking at her, you know, it was my niece… I kept on going, I didn’t want to stop” (10 May, I 2). Within the community, people share in the emotional hardship of responding to health issues among friends and family members. 

Remote context of traditional land. The themes associated with emergency response described by participants have different dimensions when experienced within, or outside, the community. There was much higher anxiety regarding emergencies that occurred away from the community; when the time to transfer to a trained health care professional is much longer, there are fewer community members present, and the environment is much more unpredictable. The remote context of the wilderness—hours and sometimes days travel from Sachigo Lake—is well known to community members. “Well, that’s traditional. They have their hunting grounds, people have different hunting grounds… There are portages, there are dangerous scenarios, anything could happen” (10 May, I 3). People commonly get stranded in remote wilderness contexts and need help from other community members. “Yeah, in the winter especially at least two or three times someone will possibly be stranded, broke down, or out of gas between here and Muskrat (Dam First Nation–over 60 km away)” (10 May, I 1).

Community members go out on-the-land as a form of subsistence living, and as a sacred way of reflecting their spirituality. Locals do not travel in these areas as a means of thrill seeking or adventure. The isolation is not perceived as dangerous, rather matter-of-fact, something familiar and embraced. This way of life does, however, present challenges in accessing help during an emergency. They do not have the luxury of waiting for trained professionals to arrive, fully-equipped to provide care. When people are on-the-land outside Sachigo Lake, they are in some of the most remote territory on the planet, but they are also at home. Community members expressed the need to adapt to their surroundings and be prepared to improvise with materials found around them when faced with a condition with potential adverse outcome.

Those in the wilderness are on their own to provide prehospital care, since a successful search and rescue is improbable and untimely. If community members do not return by the expected time, the community immediately begins planning a search and rescue, which normally includes members of the Canadian Rangers (a subcomponent of the Canadian Armed Forces reserve). “When we dispatch a search and rescue, there’s a lot of planning and by the time we deploy the group that are going on the search, maybe it’ll take more than two or three hours” (6 May, I 2). Over the hours or days before help arrives, those stranded are on their own to provide emergency care. Community members reflected on the challenges of finding assistance from people who have some form of training. People often rely on the Canadian Rangers; however, they are not always available when needed. “People who are in the Rangers are often gone all summer long and let’s face it, that’s when most of the accidents happen…” (7 May, I 2).

### 3.3. Medical Emergency

Participants of the study described locally occurring emergencies in their discussion of the health adversity faced by community members. They described a compelling sense of confidence and safety in remote wilderness locations away from the community, with an acute awareness that seemingly small issues can translate into a serious medical emergency. “We were travelling on the river dealing with rapids and all that, anything could have happened. But… going on there we were sure of ourselves if anything happened we could handle it” (6 May, I 2). “House fire or maybe somebody got hurt out on the land and asked for help. Those are serious emergencies I’m talking about, but if a kid breaks their wrist, arm on the playground, it’s a different kind of emergency. That’s within the community, we have access to the health facility” (6 May, I 2). What seemed to define an emergency included three interrelated factors: sense of isolation, condition with potential adverse outcome, and a need for help.

## 4. Discussion

Remote Indigenous communities and reserves are located on or near the hunting, fishing, and trapping grounds that are the traditional territory of families who have lived there since time immemorial. In this context, developing appropriate local emergency services generates unique cultural, epidemiological, infrastructural, and geographical challenges. While remote populations should have access to excellent emergency medical care at a standard enjoyed by all Canadians, the mechanism through which that care is delivered must be adapted to the priorities, perceptions, and needs of remote Indigenous communities. Conventional urban approaches based on ambulances, centralized dispatch, and professional paramedics may be poorly suited and financially unviable in these settings, and thus have not been included among essential local health care services. CBEC training for community members is a means of ensuring these communities have the local capacity to respond to health emergencies and manage the situation when help is hours to days away. This study provides the first exploration into the perceptions and understandings of people in this remote Indigenous community with respect to medical emergencies and the informal response system used to manage them. 

**Figure 2:**
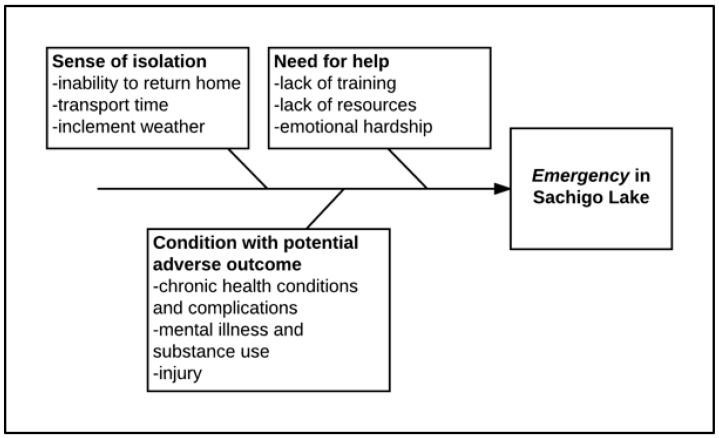
Framework for local perception of an emergency.

A framework of what constitutes a medical emergency was developed through utilizing principles of realist evaluation in analyzing the qualitative data. Realist evaluation emphasizes the need to understand the broader social and cultural context the SLWEREI was operating within to better understand the nature of emergencies in the community [34]. It was imperative to understand what an emergency means in the local context to develop an emergency response program that was locally-appropriate and meaningful. Local perceptions of health adversity and response shared three unifying features that were specific to the local context. A medical emergency in the remote context of Sachigo Lake consists of three features: (1) a sense of isolation; (2) a condition with a potentially adverse outcome; and (3) a need for help (Figure 2).

The first defining feature of locally occurring emergencies is variable perceptions of isolation. The community members of Sachigo Lake are at home in the remote context of the wilderness, but also astutely aware of the potential danger this creates if an emergency were to occur both within and away from the community. The community of Sachigo Lake is isolated from definitive hospital care and professional emergency services. This isolation increases as one travels further away from the community, such that a seemingly non-life-threatening issue, such as a sprain or fracture, or even engine problems, can translate into an inability to return home and thus a threat to health and life. The themes associated with emergency response described by participants have different dimensions when experienced within, or outside, the community. There seemed to be much higher concern regarding emergencies that occur away from the community when the time to transfer to a trained health care professional is longer, there are fewer community members present, and the environment is much more uncontrolled. Despite the awareness that community members have of the potential difficulty of returning home and obtaining assistance, there is no trepidation in going out on the land. Community members are raised in this environment, feel a connection to the area, and have a sense of wellbeing in the wilderness environment. 

The second feature of what makes an emergency is a health condition with a potential adverse outcome that requires some form of intervention. Prominent themes related to health emergency included an inability to manage chronic conditions and a fear of complications occurring away from community. There was also a high prevalence of mental illness (including substance use) and a limited local capacity for mental health response, and a high prevalence of injuries and complications of injuries while away from the community. The experiences of community members support most of the national and provincial trends concerning the disproportionate burden of health conditions experienced by Indigenous peoples such as diabetes, heart disease, mental illness, and traumatic injury [22,23,24]. This burden is exacerbated by the added complication of the remote context of the subarctic region. 

The third feature of locally occurring emergencies is the need to mobilize often under-trained and personally conflicted community members and resources. The role of care provider is challenging, since the patient is often a friend or family member in the close-knit community [9]. Community members share in the emotional hardship of responding to health issues among loved ones—witnessing their suffering and dealing with the stress of not knowing how to help. While away from the community, community members must manage these situations themselves, often untrained and ill-equipped, using only what they have around them. The need for help and sense of isolation in this context is even more compelling when compared to the regulated Response Time Standard that is measured in minutes for land ambulance dispatch in most jurisdictions of Ontario [36].

It was imperative to understand community members’ experience with health adversity to tailor curriculum and pedagogy of the SLWEREI course to meet local needs. The development of local emergency care and management systems must be informed by local notions of what an emergency is to enhance resilience and improve capacity to respond to these situations [14]. The remote nature of Sachigo Lake influences how community members understand emergencies, such that local notions of health emergencies have more to do with location and available resources than medical complexity. The guiding principles of the SLWEREI aligned with these local notions through an emphasis on local geography and community health needs, as well as using locally appropriate materials and methodology [12]. This is in stark contrast to the underlying pedagogy of conventional first aid courses, which assume that a formal response system can be activated and there will be adequate resources available to address the situation [9,37]. Furthermore, the evacuation medicine ethos of conventional wilderness first aid courses is tailored to individuals or groups who are recreating, rather than living, in remote environments. Incorporating community input and matching course content to the local context were central features of what made the SLWEREI more locally appropriate than other standardized first aid courses. The foundational learnings from the SLWEREI experience led to the exploration and emergence of CBEC as a conceptual system that better reflects the need of remote First Nation communities in northern Ontario [3,4,38].

### Limitations

The knowledge generated from this study is limited in scope and generalizability. The results reflect the perceptions of a limited sample from a single community and may not be generalizable to other remote Indigenous communities. Despite this, there is potential for transferability of the findings and resulting framework to other communities in similar circumstances. There was also a death in the community during the second SLWEREI course and many community members were in a state of mourning. The community is small and close-knit, and everyone was impacted by this event, which may have resulted in fewer participants being available to attend and share their knowledge. Nonetheless, the sample size (n = 24) and diversity does mitigate this somewhat.

There were also limitations to this study stemming from logistical issues and complex power relations. Isolated and resource-limited Indigenous communities have a compelling need for health research interventions that are locally-appropriate; however, geographical distance, challenging and costly travel, and uncertainties associated with climate make close partnerships with remote communities the most difficult to maintain [39]. The logistical constraints and limited time in the community hindered the feasibility of truly gaining an appreciation of the day-to-day nature of life in the community. However, key community champions and strong relationships built over years ensured continuity and sustainability of the program. Co-author J.B. is a local community member and health director, and has been a pivotal part of the development and success of the program—he continues to collaborate with us to expand CBEC across his tribal council and other Nishnawbe Aski Nation communities.

Research initiatives with Indigenous communities involve complex power relationships related to the history of paternalistic and often exploitive relationships between Indigenous communities and both government and research institutions [31]. Cho and Trent have highlighted authority, power, and privilege as a potential threat to validity [40], and other researchers have described the influence of this power dynamic in similar Indigenous health research contexts [41,42]. The SLWEREI team was aware of the potential power differential throughout the project and aimed to mitigate this through adhering to a community-based participatory research approach and using principles of realist evaluation, which valued community members as experts regarding their local context. The research and development of the SLWEREI began in 2009, and relationships and trust built over time helped mitigate historical power structures.

## 5. Conclusions

This study has generated a greater understanding of the local emergency health context of one remote Indigenous community in northwestern Ontario, Canada, and the hardships faced by people who have no access to paramedic or professional emergency services. Community members shared their account of the local informal response system and the types of health emergencies faced. Themes of inability to manage chronic conditions and fears of exacerbations, lack of capacity for addressing mental illness, and high prevalence of injury for community members emerged as prominent aspects of health adversity. There was also an important distinction in the experiences of community members within and outside the community, with a much higher level of anxiety for health issues that arise in remote contexts away from the community. A three-point framework of what constitutes local perceptions of an emergency emerged from the findings in this study: (1) a sense of isolation; (2) a condition with a potentially adverse outcome; and (3) a need for help. 

It is essential for local emergency care and management systems to be developed based on local perceptions of medical emergencies. Community-based training programs, such as CBEC, attend to local perceptions of emergency situations to reduce the sense of peril in the situation and enhance resilience in terms of the response capacity of community members. What community members of Sachigo Lake perceive as an emergency is much different from what may be perceived as an emergency in a more urban, well-serviced area. For members of Sachigo Lake First Nation, a medical emergency involves isolation from professional medical services in conjunction with a situation that requires assistance from known responders, and the necessity to make-do with what is available. This is contrasted with a more urban context where an emergency has more to do with activating the professional response system and awaiting assistance from unknown responders.

Existing training initiatives through the First Nations Emergency Response Program are infrequent, inconsistent, and often unavailable [37]. Conventional first aid training programs are inappropriate for remote contexts that do not have formal response services [9]. The community-based approach of the SLWEREI was effective in developing a locally appropriate and meaningful capacity development program [14]. This led to the emergence of CBEC [4], a platform that may improve medical care by providing the means to address the emergency health inequalities of the remote region of northern Ontario.

The knowledge gained from this study helps to address the lack of health-related studies concerning remote Indigenous communities in Ontario, especially related to prehospital care [3,26]. The results of this study are context-specific; however, the findings may be relevant for other remote Indigenous communities. Additional research is required to explore the health status of people in the region to find locally appropriate ways of addressing health inequities in pre-nursing station care and the incomplete chain of survival for acute health emergencies. This type of research is particularly important because of the dearth of information available, contrasted with the importance of acute care in a community that has elevated rates of chronic disease, mental illness, and traumatic injury. Future research should also assess whether CBEC programs have any impact on prevention and health promotion through encouraging the development of a safe and healthy community.

## Figures and Tables

**Table 1:** Characteristics of Sachigo Lake First Nation.

**Location**	Approximately 425 km North of Sioux Lookout Ontario in the Subarctic Region of the Northern Hemisphere
**Population** [35]	Registered population of 945
	On-reserve population of 519
**Access**	Year-round airport
	Seasonal winter road
**Medical services in community**	No formal paramedical services
	Nursing station staffed by 2–3 nurses and community health workers
	Physician services provided 3–5 days per month in community
**Travel time to hospital care**	Minimum 4 h by air ambulance in ideal weather and logistical circumstances

**Table 2:** Interview and focus group question guides and concluding survey.

Interview Guide	Focus Group Guide	Concluding Survey
What do you think the community needs to improve health?	Is the course relevant and appropriate to the community?	Did you like this course? Why or why not?
Have you been involved in a first aid situation?	What components of the course are working? What is not working?	Do you feel that you have been able to provide input into the course? Did that improve it?
Do you know of any other first aid situations in the community?	How can we improve?	Was this course different from other courses? How?
How does a medical training course affect the community?	Can you share any past experiences with the topics you have learned today?	Do you think that this course is better for the community than other courses?
Have participants shared their knowledge and skills with others?	How would what you have learned influence that situation?	
Do you think that this course meets the needs of the community?	What did you like/dislike about this section of the course?	
